# Association between high-density lipoprotein and functional outcome of ischemic stroke patients in a Taiwanese population

**DOI:** 10.1186/s12944-024-02265-z

**Published:** 2024-08-29

**Authors:** Ting-Chun Lin, Chun-Yao Huang, Yu-Ling Li, Hung-Yi Chiou, Chaur-Jong Hu, Jiann-Shing Jeng, Sung-Chun Tang, Lung Chan, Li-Ming Lien, Huey-Juan Lin, Chu-Chien Lin, Yi-Chen Hsieh

**Affiliations:** 1 Department of Neurosurgery, Hokkaido University, Sapporo, Japan; 2 Division of Cardiology and Cardiovascular Research Center, Department of Internal Medicine, Taipei Medical University Hospital, Taipei, Taiwan; 3 Taipei Heart Institute, Taipei Medical University, Taipei, Taiwan; 4 Institute of Population Health Sciences, National Health Research Institutes, Miaoli County, Taiwan; 5 School of Public Health, College of Public Health, Taipei Medical University, Taipei, Taiwan; 6 Department of Neurology, Shuang Ho Hospital, Taipei Medical University, New Taipei City, Taiwan; 7 School of Medicine, College of Medicine, Taipei Medical University, Taipei, Taiwan; 8 Taipei Neuroscience Institute, Taipei Medical University, Taipei, Taiwan; 9 Department of Neurology, National Taiwan University Hospital, Taipei, Taiwan; 10 Department of Neurology, Shin Kong Wu Ho-Su Memorial Hospital, Taipei, Taiwan; 11 Department of Neurology, Chi-Mei Medical Center, Tainan, 71004 Taiwan; 12 Ph.D Program of Neural Regenerative Medicine, College of Medical Science and Technology, Taipei Medical University, Taipei City, Taiwan

**Keywords:** Epidemiology, Cholesterol, Prognosis, Restricted cubic spline regression, *ABCA1*

## Abstract

Despite recent findings indicating a paradoxical association between high-density lipoprotein cholesterol (HDL-C) levels and cardiovascular disease (CVD) mortality, the impact of HDL-C on subsequent outcomes after ischemic stroke remains unclear. The study aims to investigate the relationships between HDL-C levels and post-stroke functional outcomes while examining the potential modifying influence of HDL-C-related single nucleotide polymorphisms identified through genome-wide association studies. This cohort study included 1,310 patients diagnosed with acute ischemic stroke (AIS), all of whom had their admission serum lipid profile and genotyping information. Participants were categorized into four groups based on gender and HDL-C level. Prognostic outcomes were assessed using a modified Rankin Scale (mRS) at 1, 3, and 12 months post-admission. Multivariate logistic regression and restricted cubic spline regression analysis were used to assess the associations between HDL-C levels and outcomes. The mean age of patients was 61.17 ± 12.08 years, and 69.31% were men. After adjusting confounders, patients with the highest HDL-C level group had a significantly higher risk of poor functional outcomes at 1, 3, and 12 months following stroke compared to the reference group. Restricted cubic splines depicted a nonlinear association between HDL-C levels and poor prognosis in both men and women. The *ABCA1* gene rs2575876 AA genotype combined with abnormal HDL-C levels exhibited a significantly heightened risk of post-stroke adverse outcomes at 1 and 3 months compared to patients with normal HDL-C levels and GG + GA genotype. These findings suggest that the combined effects of *ABCA1* genetic variants with either low or high HDL-C levels could further heighten this risk.

**Supplementary Information:**

The online version contains supplementary material available at 10.1186/s12944-024-02265-z.

## Introduction

Cerebrovascular disease continues to be the second leading cause of death and represents a major global health burden [1]. Understanding specific markers of stroke outcomes, including risk factors like hypertension or diabetes, allows healthcare professionals to tailor treatment strategies more effectively, potentially leading to better outcomes and reduced stroke-related morbidity and mortality [2].

Dyslipidemia is also widely acknowledged as a significant risk factor for adverse outcomes in cardiovascular diseases (CVD) [3]. The American Heart Association/ American Stroke Association guidelines underscore the critical role of lipid management in controlling vascular risk factors for secondary stroke prevention [4]. Nevertheless, results from the National Health and Nutrition Examination Survey spanning 2009 to 2020 indicate that lipid control rates among stroke survivors remain suboptimal [5]. Several studies have elucidated contradictory relationships between cholesterol, low-density lipoprotein cholesterol levels, and prognostic consequences in stroke patients [6, 7].

Similarly, while high-density lipoprotein cholesterol (HDL-C) has conventionally been deemed protective against CVD [8–10], recent research has uncovered a paradoxical association between HDL-C levels and CVD mortality, suggesting a U-shaped relationship [11–13]. In particular, comparable results were observed in the association between HDL-C levels and mortality from ischemic stroke [11]. Moreover, regarding the study of post-stroke outcomes, numerous previous studies have identified an inverse relationship between HDL-C levels and stroke recurrence as well as vascular complications [14–17]. In addition, countless large-scale randomized controlled trials have found that patients did not experience apparent benefits from the lipid-control treatment for CVD, even if they substantially increased HDL-C levels [18]. Mendelian randomization studies, which use genetic variants related to elevated HDL-C levels as a proxy for cumulative HDL-C exposure, have also been unable to confirm a causal relationship between HDL-C levels and CVD risk [19]. The findings imply that the influence of HDL-C on CVD mortality and related outcomes is complex, sparking further investigation into this intriguing paradox. Additionally, studies have demonstrated that genes involved in HDL-C metabolism, particularly those encoding various enzymes, may affect HDL-C levels [20–23].

Since the effect of HDL-C on outcomes following a stroke is not well understood and genes play a role in HDL-C metabolism, this study aimed to elucidate the impact of HDL-C on outcomes in acute ischemic stroke (AIS) by enrolling patients from a large nationwide registry dataset. In addition, the study explored the potential modifying role of genetic factors in the association between HDL-C levels and the risk of unfavorable outcomes among ischemic stroke patients.

## Materials and methods

### Study design and population

The Formosa Stroke Genetic Consortium (FSGC) is a collaborative research initiative that commenced in 2005, focusing on the molecular aspects of cerebrovascular diseases. The operational procedures of FSGC have been outlined in previous studies [24]. Briefly, the consortium involved ten hospitals working together to enroll cases using a standardized method, which included administering questionnaires and collecting biospecimens. Data on preadmission, inpatient clinical information, and discharge records were meticulously gathered by trained assistants or study nurses, with quality assurance measures adhering to the standards set by the Taiwan Stroke Registry (TSR) [25]. The study participants were all verified on brain computed tomography (CT) or magnetic resonance imaging (MRI) and were followed at 1, 3, and 12 months. Between 2005 and 2019, 1,310 first-ever AIS patients with genotyping data were included in this study. More than 80% of patients completed the evaluation of the functional outcomes at 1, 3, and 12 months throughout the 1-year follow-up period (supplementary Figure S1).

Since the guidelines of HDL-C from the National Cholesterol Education Program III for Asians were less than 40 mg/dL in men or less than 50 mg/dL in women [26] and few Taiwanese populations have extremely high HDL-C concentrations [27], the study participants were stratified into four groups relying on gender and HDL-C level. Group I was characterized by HDL-C levels below 0.78 mM/L for males and below 1.04 mM/L for females. Group II comprised individuals with HDL-C levels ranging from 0.78 mM /L to less than 1.04 mM /L for males and from 1.04 mM /L to less than 1.30 mM /L for females. Group III served as the reference group, with HDL-C levels between 1.04 mM /L and less than 1.30 mM /L for males and between 1.30 mM /L and less than 1.55 mM /L for females. Group IV consisted of participants with HDL-C levels equal to or greater than 1.30 mM /L for males and equal to or greater than 1.55 mM /L for females.

This study received approval from the Institutional Review Board or Ethical Committees at Taipei Medical University and all collaborative hospitals. Written informed consent was obtained from all study subjects or their relatives, ensuring that ethical standards and participants’ rights were respected throughout the research process.

### Data collection

During the admission process, demographic characteristics, medical history, medication usage history, and clinical features were systematically gathered using a standardized registry form. To determine the severity of the stroke at the beginning, trained neurologists utilized the National Institute of Health Stroke Scale (NIHSS). Additionally, within 24 h of the onset of AIS, fasting glucose levels, glycated hemoglobin (HbA1c), total cholesterol, triglyceride levels, low-density lipoprotein cholesterol (LDL-C), and HDL-C were measured in each participating hospital. This comprehensive approach ensured the collection of essential data for the study and facilitated the evaluation of various stroke-related factors.

### Outcome measures

The modified Rankin Scale (mRS), a standard tool for assessing functional outcomes, was employed to evaluate the prognosis of stroke patients at 1, 3, 6, and 12 months post-stroke. The scale ranges from 0 to 6, with 0 indicating no symptoms, 5 signifying severe disability, and 6 representing death. In this study, patients with an mRS score of 0 to 2 were classified as having favorable outcomes, while those with a score of 3 or higher were categorized as having unfavorable outcomes.

### Selection of HDL-C-related SNP

In this study, HDL-C-related single nucleotide polymorphisms (SNPs) were initially identified through a genome-wide association study (GWAS) analysis of HDL-C, using a cohort of 45,575 individuals from the Taiwan Biobank, with 36.31% being males and an average age of 49.21 ± 10.99 years (supplementary Table S1). In brief, Taiwan Biobank is a prospective cohort study offering extensive phenotypic and genetic data for the Taiwanese population. Genotyping was carried out using the TWBv1 array and TWBv2 array, following a standardized quality control pipeline and imputation protocols [28]. A Q-Q plot illustrated the evaluation of SNP enrichment for HDL-C levels, and significant genetic loci were depicted in a Manhattan plot (supplementary Figure S2). Notably, 15 SNPs were identified on chromosome 9, and chromosome 16 reached a significant level (*P*-value = 5*10^− 8^), as detailed in supplementary Table S2. Subsequently, only 9 SNPs associated with HDL-C from the GWAS catalog database (https://www.ebi.ac.uk/gwas/) were selected, specifically those in cholesteryl ester transfer protein (*CETP*) and ATP-binding cassette A1 (*ABCA1*) genes.

### Genotyping

Extraction of genomic DNA from the buffy coat fractions was adopted by a non-organic purification method and stored at -80℃ until genotyping. Genotyping was conducted with the Axiom Genome-Wide TWB 2.0 Array Plate from the National Center for Genome Medicine in Taiwan. Samples were excluded if the call rate was less than 98%. Genotype imputation was used by the Michigan Imputation Server (https://imputationserver.sph.umich.edu) utilizing the 1000G Phase 1 version 3 reference panel [29]. Quality control and filtering were carried out using PLINK software. The variants were mapped by adopting the GRCh37/hg19 reference genome coordinates, and phasing was executed using the Eagle v2.4 algorithm.

### Statistical methods

The mean difference among four HDL-C groups was performed using the ANOVA method with Scheffe post hoc analysis, while the frequency variation was examined using the Chi-square test. Multivariate logistic regression models were employed to estimate the relationship between HDL-C groups and functional outcomes at 1, 3, and 12 months following a stroke. Odds ratio (OR) and 95% CI were determined for each HDL-C group relative to the reference group (Group III). Conventional risk factors for the prognosis of ischemic stroke, including age, gender, body mass index (BMI), initial stroke severity, hypertension, diabetes mellitus, tobacco smoking, and alcohol consumption, were considered covariates. To visualize the non-linear relationship between HDL-C level and adverse outcomes at 1, 3, and 12 months after a stroke, a restricted cubic spline (RCS) regression model was used with four knots based on gender-specific criteria. All statistical analyses were performed using SAS (version 9.4, Cary, North Carolina) and R (version 4.3.1) statistical software. A two-tailed *P* value of less than 0.05 was considered statistically significant.

## Results

### Baseline characteristics

The baseline characteristics of the study participants categorized into different HDL-C groups are shown in Table 1. Among 1,310 first-ever AIS patients, the average age was 61.17 ± 12.08 years, with nearly 70% of the patients being male. Significant differences were observed in the average age, BMI, serum HbA1c level, lipid profiles, initial stroke severity, and the frequencies of gender, hypertension, and diabetes mellitus histories, cigarette smoking, alcohol consumption, lipid-lowering drugs, and anti-diabetics medicine across the four HDL-C groups.

**Table 1:** Baseline characteristics among ischemic stroke patients with different HDL-C level

	TOTAL (*N* = 1310)	Group I (*N* = 197)	Group II (*N* = 464)	Group III (*N* = 378)	Group IV (*N* = 271)	*P*-value
Age, _year, mean±SD_	61.17 ± 12.08	62.43 ± 11.75^ab^	60.45 ± 11.37^ac^	59.88 ± 12.62^c^	63.30 ± 12.44^b^	**0.0009**
SEX, _n(%)_						
Female	402(30.69)	106(53.81)	129(27.80)	79(20.90)	88(32.47)	**< 0.0001**
Male	908(69.31)	91(46.19)	335(72.20)	299(79.10)	183(67.53)	
BMI, _kg/m2, mean±SD_	25.77 ± 3.98	25.88 ± 4.10^a^	26.09 ± 3.97^a^	26.13 ± 4.05^a^	24.62 ± 3.60^b^	**0.0003**
SBP, _mmHg, mean±SD_	166.27 ± 31.63	163.19 ± 29.26	166.81 ± 31.55	165.49 ± 31.95	168.71 ± 32.90	0.2801
DBP, _mmHg, mean±SD_	96.55 ± 20.99	94.72 ± 19.40	96.74 ± 20.98	97.59 ± 21.44	96.12 ± 21.50	0.4627
HbA1c, _%, mean±SD_	7.13 ± 2.71	7.71 ± 4.86^a^	7.18 ± 2.04^b^	6.95 ± 2.10^b^	6.86 ± 2.18^b^	**0.0039**
Fasting glucose, _mM/L, mean±SD_	7.27 ± 2.98	7.78 ± 3.17	7.16 ± 2.67	7.11 ± 3.12	7.29 ± 3.12	0.0719
Cholesterol, _mM/L, mean±SD_	5.07 ± 1.27	4.68 ± 1.25^a^	4.89 ± 1.09^b^	5.19 ± 1.30^c^	5.51 ± 1.38^d^	**< 0.0001**
Triglyceride, _mM/L, mean±SD_	1.86 ± 1.50	2.31 ± 1.53^a^	2.00 ± 1.47^b^	1.76 ± 1.62^c^	1.42 ± 1.17^d^	**< 0.0001**
LDL-C, _mM/L, mean±SD_	3.32 ± 1.02	3.02 ± 1.04^a^	3.15 ± 0.9^a^	3.36 ± 1.01^b^	3.3 ± 1.19^b^	**0.0003**
NIHSS at beginning, _score, mean±SD_	5.19 ± 5.10	5.20 ± 4.62^a^	4.74 ± 4.40^a^	5.00 ± 5.25^a^	6.18 ± 6.09^b^	**0.0024**
TOAST, _n(%)_						
LAA	349(30.11)	56(32.94)	128(30.77)	104(31.71)	61(24.90)	0.1510
SVO	598(51.60)	78(45.88)	228(54.81)	157(47.87)	135(55.10)	
CE	107(9.23)	17(10.00)	26(6.25)	34(10.37)	30(12.24)	
SE	14(1.21)	3(1.76)	6(1.44)	4(1.22)	1(0.41)	
UE	91(7.85)	16(9.41)	28(6.73)	29(8.84)	18(7.35)	
Heart disease, _n(%)_	283(21.62)	48(24.37)	85(18.32)	87(23.02)	63(23.33)	0.1900
Dyslipidemia, _n(%)_	776(59.46)	121(62.05)	275(59.40)	224(59.42)	156(57.78)	0.8348
Hypertension, _n(%)_	1037(79.22)	169(86.22)	369(79.53)	287(75.93)	212(78.23)	**0.0364**
Diabetes mellitus, _n(%)_	558(42.69)	106(54.08)	204(44.16)	152(40.21)	96(35.42)	**0.0005**
Cigarette smoking, _n(%)_	653(50.00)	74(37.76)	253(54.64)	206(54.79)	120(44.28)	**< 0.0001**
Alcohol drinking, _n(%)_	237(18.15)	18(9.18)	81(17.49)	87(23.14)	51(18.82)	**0.0007**
Lipid-lowering drug, _n(%)_	889(67.86)	120(60.91)	332(69.40)	272(71.96)	175(64.58)	**0.0278**
Anti-hypertensive drug, _n(%)_	746(56.95)	115(58.38)	269(57.97)	203(53.70)	159(58.67)	0.5098
Anti-diabetics drug, _n(%)_	470(35.88)	88(44.67)	177(38.15)	125(33.07)	80(29.52)	**0.0033**

Subjects were divided into groups according to sex and HDL-C level. Group I (M: HDL-C < 0.78 mM/L, F: HDL-C < 1.04 mM/L); Group II (M: 0.78 mM/L ≦ HDL-C < 1.04 mM/L, F: 1.04 mM/L ≦ HDL-C < 1.30 mM/L); Group III as a reference group (M: 1.04 mM/L ≦ HDL-C < 1.30 mM/L, F: 1.30 mM/L ≦ HDL-C < 1.55 mM/L); Group IV (M: HDL-C ≧ 1.30 mM/L, F: HDL-C ≧ 1.55 mM/L)

BMI, Body Mass Index; SBP, Systolic Blood Pressure; DBP, Diastolic Blood Pressure; HDL-C, High Density Lipoprotein Cholesterol; LDL-C, Low Density Lipoprotein Cholesterol; LAA, Large Artery Atherosclerosis; SVO, Small Vessel Occlusion; CE, Cardioembolism; SE, Specific etiology; UE, Undetermined etiology

Groups denoted with different letters (a, b, c, d) indicate statistically significant differences using Scheffe post hoc analysis

### Association between HDL-C level and functional outcomes

**Fig. 1:**
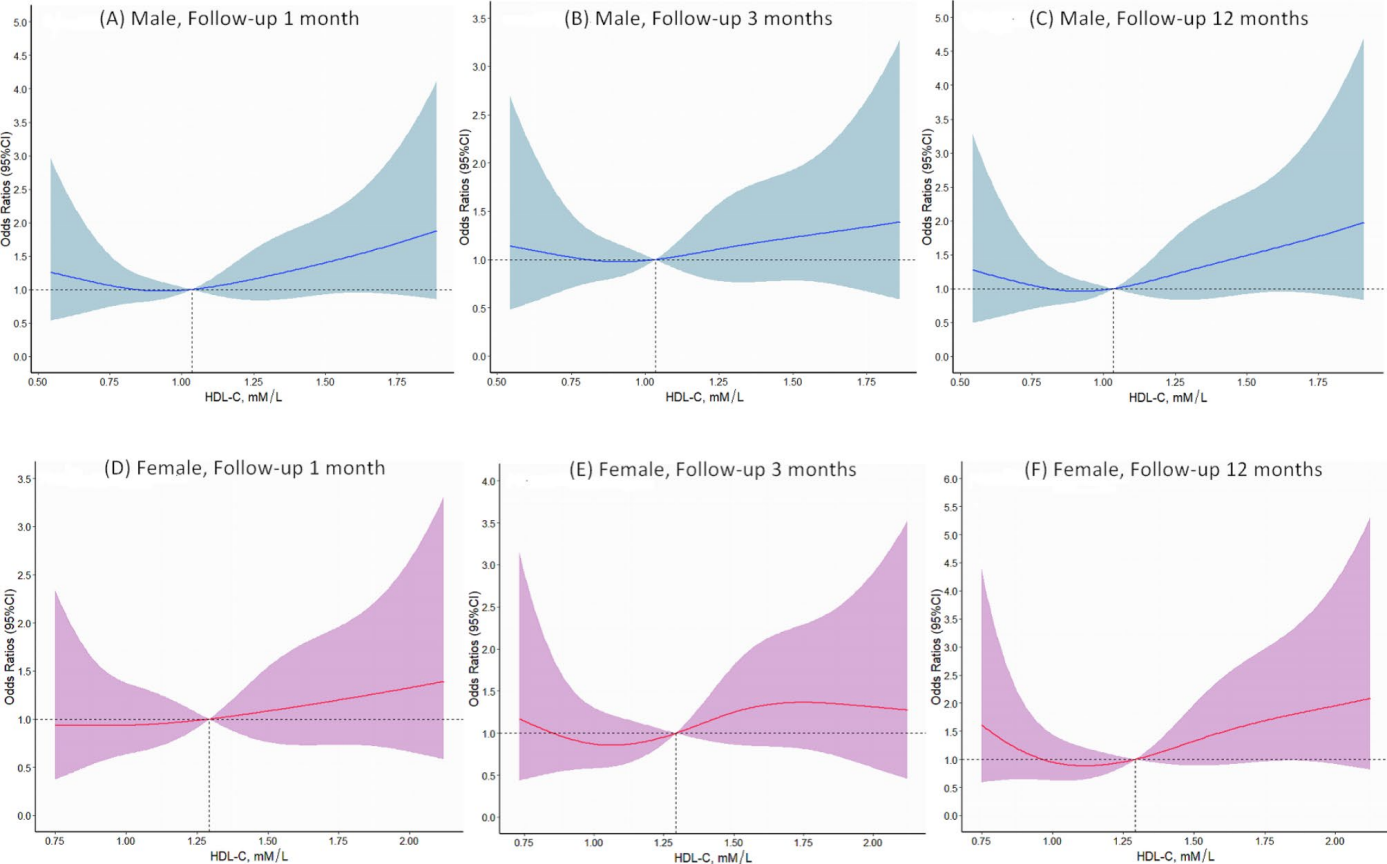
Odds ratios (ORs) for unfavorable outcomes of ischemic stroke patients at 1, 3, and 12 months according to different levels of HDL-C after adjusting for age, gender, BMI, initial stroke severity, hypertension, diabetes, smoking, alcohol drinking, lipid-lowering drug, and anti-diabetics drug

The association between four different HDL-C level groups with unfavorable outcomes at 1, 3, and 12 months is presented in Table 2. Patients in Group I and II had worse neurological outcomes at 1, 3, and 12 months compared with Group III, which served as the reference group; however, these differences did not achieve statistical significance, except for women in Group II at 1-month follow-up (OR, 2.17; 95%CI, 1.02–4.64). Notably, patients in Group IV had a significantly increased risk of adverse outcomes at 1, 3, and 12 months compared with the reference group, regardless of the overall population or gender, apart from male patients followed at three months. Furthermore, the RCS plot for analyzing the relationship between HDL-C level and the risk of poor outcomes at 1, 3, and 12 months after stroke stratified by gender was exhibited in Fig. 1. The shape of the dose-response correlation between HDL-C level and unfavorable prognosis was non-linear in both men and women.

**Table 2:** Association between different HDL-C level groups and unfavorable outcomes at 1, 3, and 12 months

		Total					Females					Males			
Outcomes	HDL-C	<=2	>=3	OR^a^ (95%CI)	*P*-value		<=2	>=3	OR^a^ (95%CI)	*P*-value		<=2	>=3	OR^a^ (95%CI)	*P*-value
**1 month**	Group I	128(14.40)	47(15.02)	1.59(0.98–2.57)	0.0595		60(25.00)	30(24.00)	1.95(0.86–4.41)	0.1091		68(10.48)	17(9.04)	1.22(0.61–2.41)	0.5758
	Group II	318(35.77)	105()33.55	1.44(0.98–2.12)	0.0638		77(32.08)	43(34.40)	**2.17(1.02–4.64)**	**0.0451**		241(37.13)	62(32.98)	1.18(0.75–1.87)	0.4787
	Group III	279(31.38)	75(23.96)	1.0			57(23.75)	18(14.40)	1.0			222(34.21)	57(30.32)	1.0	
	Group IV	164(18.45)	86(27.48)	**1.95(1.28–2.98)**	**0.0018**		46(19.17)	34(27.20)	**2.47(1.10–5.53)**	**0.0288**		118(18.18)	52(27.66)	**1.78(1.08–2.95)**	**0.0241**
**3 months**	Group I	129(14.24)	41(15.83)	1.51(0.90–2.55)	0.1193		62(24.90)	27(23.89)	1.81(0.78–4.20)	0.1658		67(10.20)	14(9.59)	1.06(0.48–2.31)	0.8885
	Group II	326(35.98)	84(32.43)	1.34(0.88–2.05)	0.1717		81(32.53)	37(32.74)	1.81(0.83–3.98)	0.1378		245(37.29)	47(32.19)	1.13(0.68–1.89)	0.6366
	Group III	278(30.68)	63(24.32)	1.0			58(23.29)	17(15.04)	1.0			220(33.49)	46(31.51)	1.0	
	Group IV	173(19.09)	71(27.41)	**1.76(1.12–2.78)**	**0.0143**		48(19.28)	32(28.32)	**2.46(1.07–5.68)**	**0.0351**		125(19.03)	39(26.71)	1.56(0.90–2.73)	0.1158
**12 months**	Group I	121(14.40)	33(14.93)	1.65(0.93–2.93)	0.0867		58(24.79)	24(26.67)	2.31(0.93–5.77)	0.0720		63(10.40)	9(6.87)	0.80(0.30–2.10)	0.6438
	Group II	295(35.12)	70(31.67)	1.49(0.94–2.37)	0.0910		78(33.33)	28(31.11)	1.76(0.74–4.18)	0.2020		217(35.81)	42(32.06)	1.38(0.79–2.43)	0.2581
	Group III	259(30.83)	51(23.08)	1.0			53(22.65)	13(14.44)	1.0			206(33.99)	38(29.01)	1.0	
	Group IV	165(19.64)	67(30.32)	**2.24(1.38–3.63)**	**0.0012**		45(19.23)	25(27.78)	**2.66(1.05–6.75)**	**0.0396**		120(82.23)	42(32.06)	**2.27(1.27–4.08)**	**0.0060**

a: Adjustment variable: age, gender, BMI, initial stroke severity, hypertension, diabetes, smoking, alcohol drinking, lipid-lowering drug, and anti-diabetics drug

Subjects were divided into groups according to sex and HDL-C level. Group I (M: HDL-C < 0.78 mM/L, F: HDL-C < 1.04 mM/L); Group II (M: 0.78 mM/L ≦ HDL-C < 1.04 mM/L, F: 1.04 mM/L ≦ HDL-C < 1.30 mM/L); Group III as a reference group (M: 1.04 mM/L ≦ HDL-C < 1.30 mM/L, F: 1.30 mM/L ≦ HDL-C < 1.55 mM/L); Group IV (M: HDL-C ≧ 1.30 mM/L, F: HDL-C ≧ 1.55 mM/L)

### HDL-C-related SNPs and their association with HDL-C level and adverse outcomes

**Fig. 2:**
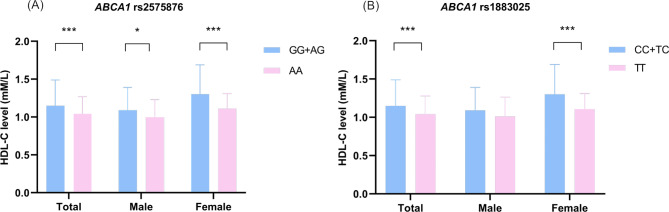
Comparison of HDL-C levels in different genotypes under recessive model for (**A**) rs2575876 and (**B**) rs1883025 SNPs in *ABCA1* gene in the total population, men and women. *: *P* < 0.05, ***: *P* < 0.001

The significant nine selected HDL-C-related SNPs and their association with serum HDL-C concentrations were illustrated in the supplementary Table S3. The analysis revealed that only two SNPs, *ABCA1* rs1883025 and rs2575876, were significantly associated with HDL-C levels in the total population and within subgroups of men and women (supplementary Figure S3). Subsequently, the association between these SNPs and adverse outcomes following stroke was investigated. The results showed that the recessive model of both rs2575876 and rs1883025 was notably associated with adverse outcomes at 1 and 3 months post-stroke onset (supplementary Table S4). Figure 2 depicts the HDL-C levels across different genotypes under the recessive model for rs2575876 and rs1883025 SNPs in the *ABCA1* gene. The findings revealed notable differences in HDL-C levels among both SNPs under the recessive model, irrespective of the total population and women, except for the results of rs1883025 among men.

### The combined effect of HDL-C level and *ABCA1* rs2575876 on the risk of worse outcomes after stroke

**Fig. 3:**
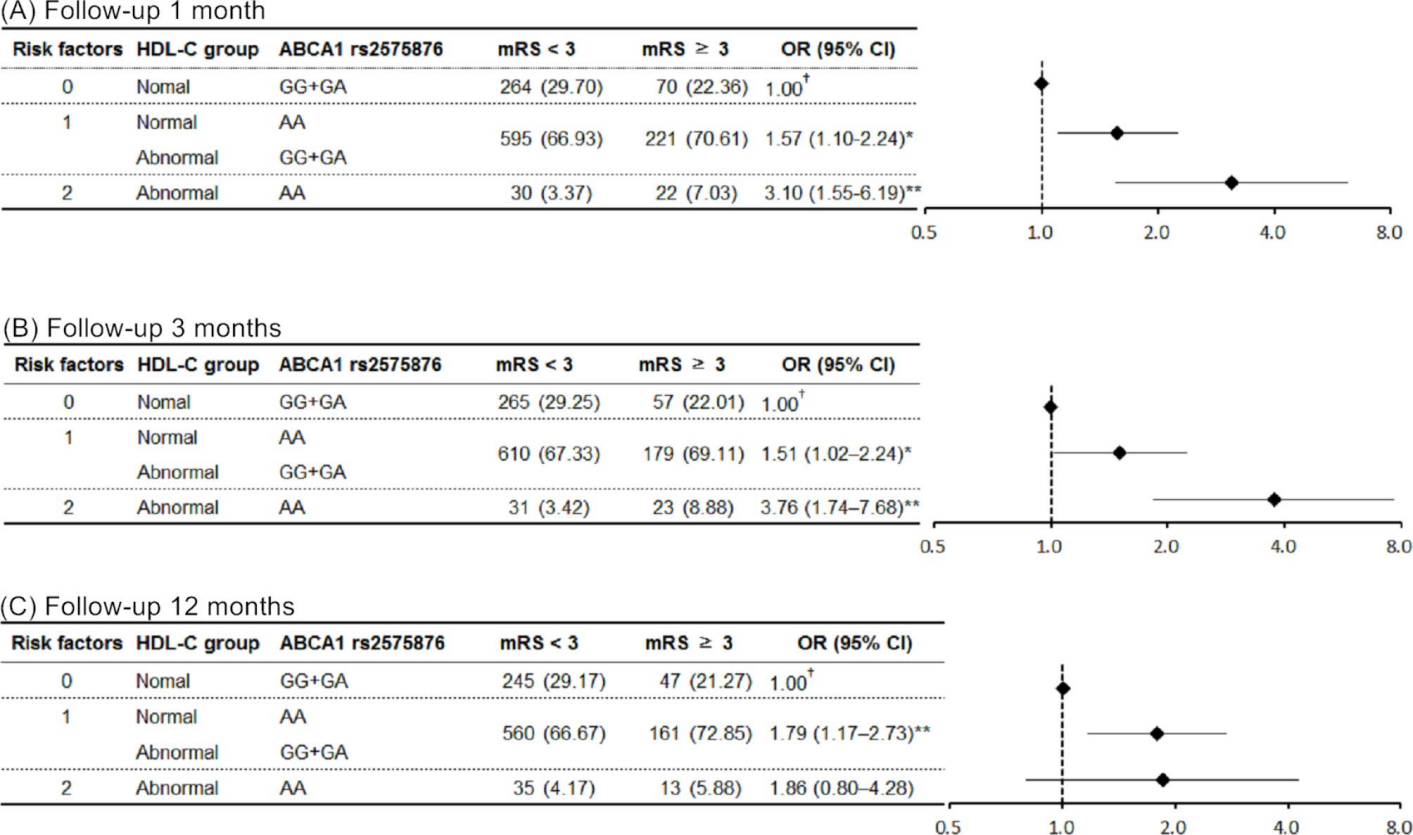
Association between combined *ABCA1* rs2575876 variants, HDL-C levels, and unfavorable outcomes following ischemic stroke. Subjects were divided into groups according to sex and HDL-C level. Normal group (M: 1.04 mM/L ≦ HDL-C < 1.30 mM/L, F: 1.30 mM/L ≦ HDL-C < 1.55 mM/L) and abnormal group (M: HDL-C < 1.04 mM/L or ≧ 1.30 mM/L, F: HDL-C < 1.30 mM/L or ≧ 1.55 mM/L); OR was adjusted by age, gender, BMI, initial stroke severity, hypertension, diabetes, smoking, alcohol drinking, lipid-lowering drug, and anti-diabetics drug. †: *P* for trend < 0.05

Due to the significant findings of HDL-C levels and *ABCA1* gene rs2575876, the combined effects on the risk of worsening prognosis after stroke were further analyzed. Figure 3 illustrates the relationship between rs2575876 SNP under the recessive model and two groups of HDL-C concentrations, categorized as normal group (1.04 ~ 1.29 mM /L for males and 1.03 ~ 1.53 mM /L for females) and abnormal group defined as low (< 1.04 mM /L for males and < 1.30 mM /L for females) and high levels (≥ 1.30 mM /L for males and ≥ 1.53 mM /L for females) on the risk of poor outcomes. The findings revealed that individuals with abnormal HDL-C levels and the AA genotype of rs2575876 exhibited a significantly heightened risk of adverse outcomes after stroke at 1 and 3 months, except for the 12 months, compared to patients with normal HDL-C levels and GG + GA genotype. However, a substantial increase in poor outcomes was observed when patients were exposed to these elevated risk factors at 1, 3, and 12 months of follow-up.

## Discussion

The finding from this multicenter registry-based study demonstrated that the serum level of HDL-C revealed a U-shape relationship with clinical functional outcomes at 1, 3, and 12 months after stroke, regardless of gender. Furthermore, individuals with abnormal HDL-C levels and the rs2575876 AA genotype in the *ABCA1* gene had a more significantly elevated risk of adverse outcomes at 1 and 3 months after stroke.

Several recent research has revealed a U-shaped relationship between HDL-C levels and CVD mortality [11–13], particularly concerning ischemic stroke. However, other studies have reported that lower serum HDL-C levels were related to worse neurological or cardiovascular outcomes following a stroke [14–17]. Previous findings indicated that HDL function could be a more accurate indicator of CVD risk than HDL-C levels alone [30]. A recent prospective study demonstrated an inverse correlation between HDL antioxidant capacity in AIS and NIHSS scores at admission, identifying capacity as an important predictor of clinical outcomes [31]. Moreover, multiple studies indicate that while normal HDL typically exhibits anti-inflammatory properties, throughout the acute phase of stroke, it may paradoxically promote inflammation, thereby transforming “good” cholesterol into “bad” cholesterol [32]. During the acute stage of ischemic stroke, levels of HDL-related proteins such as alpha-1 anti-trypsin, myeloperoxidase, and paraoxonase-1 may compromise the antioxidant capabilities of HDL [33]. Notably, myeloperoxidase, an enzyme found in high concentrations in macrophages at atherosclerotic lesions, specifically targets apolipoprotein A-1, the major protein of HDL, leading to cholesterol accumulation in macrophages [34]. Considering these findings, the apparent association between higher HDL-C levels and worse outcomes in these results may be attributed to the pro-inflammatory nature of HDL in the context of AIS.

The impact of genetic factors, including SNPs in genes encoding various enzymes, on HDL-C levels has been well-documented. Despite the intricate nature of HDL-C metabolism regulation, enzymes within the reverse cholesterol transport (RCT) system, such as ABCA1, Lecithin: cholesterol acyltransferase (LCAT), CETP, hepatic lipase, APOA1/C3/A4/A5, scavenger receptor B type I (SCARB1), and lipoprotein lipase, are known to play significant roles. From the GWAS analysis on HDL-C levels utilizing the Taiwan Biobank dataset, 9 SNPs on *CETP* and *ABCA1* genes exhibited substantial associations with HDL-C expression, also included in the database of GWAS catalog. Specifically, one SNP, rs2575876, situated on the *ABCA1* gene, demonstrated a consistent association with HDL-C levels under a recessive model across study subjects, including the total population, men and women. Furthermore, this SNP showed a remarkable association with unfavorable outcomes at 1 and 3 months post-stroke. Notably, rs2575876 has also been linked to lipid levels in previous research [35, 36] and identified as one of multiple genetic variants directly related to ischemic stroke in the Southern Chinese population [37].

The *ABCA1* gene, found on chromosome 9q31.1 [38], is widely acknowledged as a crucial cholesterol transporter, pivotal in maintaining cholesterol balance within the brain and thereby regulating cholesterol homeostasis [39]. These variants, intronic, nonsynonymous, or located in the promoter region, significantly affect ABCA1 protein function and expression[40]. Deficiency or genetic abnormalities in ABCA1 have increased the susceptibility to cerebrovascular diseases [41] and can worsen outcomes following a stroke by impairing the blood-brain barrier and white matter [42, 43]. These genetic variants in *ABCA1*, whether rare or common, can influence circulating levels of HDL-C. A recent meta-analysis highlighted that *ABCA1* polymorphisms can potentially impact plasma lipid levels, which play a role in various diseases. This underscores the potential utility of *ABCA1* genotyping in clinical settings for managing lipid profiles effectively[44].

The combined effects of abnormal HDL-C levels (both low and high HDL-C levels) and rs2575876 AA genotype of *ABCA1* gene on the risk of poor outcomes after stroke were investigated in this study. The findings revealed that the risk of unfavorable outcomes increased as risk factors increased. It was further hypothesized that the *ABCA1* gene might not only modulate the effect of lower HDL-C levels but also influence the impact of higher HDL-C expression on poor outcomes. Interestingly, the interaction analysis also reflected an additive interaction between abnormal HDL-C levels and *the ABCA1* gene in worsened outcomes (supplementary Table S5).

### Strengths and limitations

The advantages of this study include a large population-based stroke registry, standardized information collection protocols, and a relatively high follow-up rate (85%). Despite these strengths, certain limitations warrant consideration. Firstly, high HDL-C levels were defined as ≥ 1.30 mM /L in men and ≥ 1.55 mM /L in women in this study, whereas other studies defined it as > 2.07 mM /L [45, 46]. The difference in definition of high HDL-C levels was that less than 3% of patients had HDL-C levels > 2.07 mM /L, potentially limiting the generalizability of the findings to other populations. Secondly, while the HDL-C concentration measurement was only examined at baseline, changes during the follow-up period could also bear significance. Thirdly, unmeasured confounding factors may influence the observed associations despite possible covariates being considered in the multivariable regression analysis. Lastly, it is worth noting that prior findings suggest that functional measurements of HDL-C, such as particle numbers and size, have demonstrated more significant cardioprotective potential compared to HDL-C levels alone [47]. However, this study did not analyze these functional measurements, highlighting an avenue for future investigation in subsequent analyses.

## Conclusions

The results indicated a nonlinear association between HDL-C levels and poor prognosis following ischemic stroke. Genetic variants in the *ABCA1* gene may influence HDL-C expression, potentially exacerbating the risk of adverse outcomes following a stroke. The combined effects of abnormal HDL-C levels and *ABCA1* genetic variants could further heighten this risk. These findings carry significant clinical implications, suggesting that maintaining HDL-C within the normal range contributes to favorable outcomes after a stroke. This is particularly important given that HDL-C measurements are commonly utilized for assessing CVD risk and predicting stroke outcomes.

### Electronic supplementary material

Below is the link to the electronic supplementary material.

Supplementary Material 1

## Data Availability

The datasets that support the findings of this study are available from the corresponding author upon reasonable request.

## Abbreviations

CVD: Cardiovascular disease
mRS: Modified Rankin Scale
CT: Computed tomography
MRI: Magnetic resonance imaging
AIS: Acute ischemic stroke
NIHSS: The National Institute of Health Stroke Scale
HbA1c: Glycated hemoglobin
GWAS: Genome-wide association study
CETP: Cholesteryl ester transfer protein
ABCA1: ATP-binding cassette A1
OR: Odds ratio
CI: Confidence interval
RCS: Restricted cubic spline
RCT: Reverse cholesterol transport
LCAT: Lecithin: cholesterol acyltransferase, SCARB1,scavenger receptor B type I
