# Supplementary material for: Association between high-density lipoprotein and functional outcome of ischemic stroke patients in a Taiwanese population
Source: Lipids Health Dis. 2024 Aug 29;23:275. doi: 10.1186/s12944-024-02265-z (PMC11363607; doi:10.1186/s12944-024-02265-z)
Supplement: Supplementary file 1 — Supplementary Material 1 [file 12944_2024_2265_MOESM1_ESM.docx]

**Association between high-density lipoprotein and functional outcome of ischemic stroke patients in a Taiwanese population**

Ting-Chun Lin, Chun-Yao Huang, Yu-Ling Li, Hung-Yi Chiou, Chaur-Jong Hu, Jiann-Shing Jeng, Sung-Chun Tang, Lung Chan, Li-Ming Lien, Huey-Juan Lin, Chu-Chien Lin, Yi-Chen Hsieh

**Additional files**

**Table S1.** Baseline characteristics of 45,575 individuals from Taiwan Biobank dataset

**Table S2.** The information of fifteen significant SNPs and their association with HDL-C levels

**Table S3.** HDL-C levels by different genotypes of *CETP* and *ABCA1* SNPs

**Table S4.** Association between *ABCA1* and *CETP* genes and stroke outcomes assessed using mRS at 1-, 3-, and 12-months

**Table S5.** Association between combined *ABCA1* rs2575876 variants and HDL-C levels and unfavorable outcomes at 1, 3 and 12 months

**Fig S1.** Flowchart of participants recruited for this study

**Fig S2.** (A) Q-Q plot for *P* values from Taiwan Biobank GWAS database. The vertical and horizontal axes indicate observed and expected –log_10_ (*P* value) for tests of association between SNPs and HDL-C level respectively. (B) Manhattan plot (-log_10_ of the *P* value based on genomic location) of the association between the SNPs denoted in the GWAS and the HDL-C levels shows the formation of 15 SNPs over the line representing P < 5*10^-8^. GWAS, genome-wide association study; SNPs, single nucleotide polymorphisms; HDL-C, high-density lipoprotein cholesterol

**Fig S3.** Comparison of HDL-C level in each genotype of (A) rs2575876 and (B) rs1883025 SNPs in *ABCA1* gene. HDL-C, high-density lipoprotein cholesterol; SNPs, single nucleotide polymorphisms; ATP Binding Cassette Subfamily A Member 1, ABCA1; *: *P*-value<0.05

**Table S1**. Baseline characteristics of 45,575 individuals from Taiwan Biobank dataset

| **Variables** |  | ***TWB subjects***  ***(N=45,575)*** | |
| --- | --- | --- | --- |
| AGE |  | 49.21 | ± 10.99 |
| SEX | Male | 16548 | (36.31%) |
|  | Female | 29027 | (63.69%) |
| Weight (kg) |  | 64.12 | ± 13.02 |
| Waist circumference (cm) |  | 83.31 | ± 10.36 |
| Lipid profiles | Total cholesterol (mM/L) | 5.1 | ± 0.9 |
|  | Triglyceride (mM/L) | 1.3 | ± 1.1 |
|  | LDL-C (mM/L) | 3.1 | ± 0.8 |
| Alcohol drinking | None or few | 41948 | (92.04%) |
|  | Quit | 1064 | (2.33%) |
|  | Current drinking | 2563 | (5.62%) |
| Cigarette smoking | Yes | 32594 | (71.52%) |
|  | No | 12981 | (28.48%) |
| Regularly habitual exercise | Yes | 27955 | (61.34%) |
|  | No | 17620 | (38.66%) |
| Disease history | Hypertension | 5345 | (11.73%) |
|  | Dyslipidemia | 3323 | (7.29%) |
|  | Diabetes mellitus | 2213 | (4.86%) |

LDL-C, low-density lipoprotein cholesterol

**Table S2**. The information of fifteen significant SNPs and their association with HDL-C levels

| **SNP** | **Location** | **REF/ALT** | **GENE** | **Frequency of ALT** | **Association with HDL-C levels**  **Coeff ^a^** | ***P*-value** |
| --- | --- | --- | --- | --- | --- | --- |
| rs36229491 | 16:56960332 | ->A | *CETP* | 0.279 | 0.925 | 6.56E-36 |
| rs3764261^*^ | 16:56959412 | C>A | *CETP, HERPUD1* | 0.317 | 0.912 | 5.72E-35 |
| rs12149545 | 16:56959249 | G>A | *CETP* | 0.280 | 0.912 | 6.62E-35 |
| rs183130^*^ | 16:56957451 | C>T | *CETP, HERPUD1* | 0.315 | 0.909 | 1.87E-34 |
| rs247617^*^ | 16:56956804 | C>A | *CETP, HERPUD1* | 0.318 | 0.898 | 7.13E-34 |
| rs173539^*^ | 16:56954132 | C>T | *CETP, HERPUD1* | 0.330 | 0.612 | 4.17E-18 |
| rs12720926^*^ | 16:56965006 | A>C,G | *CETP* | 0.247 | 0.538 | 1.08E-15 |
| rs7205804^*^ | 16:56970977 | G>A,T | *CETP* | 0.407 | 0.505 | 7.19E-15 |
| rs11508026 | 16:56965416 | C>T | *CETP* | 0.361 | 0.512 | 7.69E-15 |
| rs12444012 | 16:56967526 | G>A,C,T | *CETP* | 0.378 | 0.512 | 8.60E-15 |
| rs4784741 | 16:56967304 | C>G,T | *CETP* | 0.378 | 0.507 | 2.18E-14 |
| rs708272 | 16:56962376 | G>A,C | *CETP* | 0.426 | 0.465 | 1.62E-13 |
| rs711752^*^ | 16:56962299 | G>A,C | *CETP* | 0.380 | 0.464 | 1.73E-13 |
| rs1883025^*^ | 9:104902020 | C>T | *ABCA1* | 0.263 | -0.555 | 1.00E-10 |
| rs2575876^*^ | 9:104903458 | G>A | *ABCA1* | 0.256 | -0.551 | 1.45E-10 |

*: The SNPs were included in GWAS catalog database. a The coefficient value represents change in HDL-C per ALT allele copy (0, 1, 2) for the SNP. REF, reference allele; ALT, alternative allele; Coeff, coefficient; HDL-C, high-density lipoprotein cholesterol; SNPs, single nucleotide polymorphisms; GWAS, genome-wide association study.

**Table S3.** HDL-C levels by different genotypes of *CETP* and *ABCA1* SNPs

|  |  | Total | | | |  | Male | | | |  | Female | | | |
| --- | --- | --- | --- | --- | --- | --- | --- | --- | --- | --- | --- | --- | --- | --- | --- |
|  |  | N | Mean | SD | *P*-velue |  | N | Mean | SD | *P*-velue |  | N | Mean | SD | *P*-velue |
| ***CETP*** |  |  |  |  |  |  |  |  |  |  |  |  |  |  |  |
| rs3764261 | CC | 921 | 43.88 | (12.90) ^a^ | **0.0087** |  | 631 | 41.39 | (11.41) ^a^ | **0.0041** |  | 290 | 49.29 | (14.24) | 0.3962 |
|  | CA | 360 | 44.66 | (13.61) ^a^ |  |  | 256 | 42.27 | (11.40) ^a^ |  |  | 104 | 50.53 | (16.56) |  |
|  | AA | 29 | 51.32 | (13.33) |  |  | 21 | 49.63 | (12.88) ^b^ |  |  | 8 | 55.75 | (14.34) |  |
| rs183130 | CC | 923 | 43.86 | (12.89) ^a^ | **0.0081** |  | 633 | 41.38 | (11.40) ^a^ | **0.0038** |  | 290 | 49.29 | (14.24) | 0.3962 |
|  | CT | 358 | 44.70 | (13.62) ^a^ |  |  | 254 | 42.32 | (11.42) ^a^ |  |  | 104 | 50.53 | (16.56) |  |
|  | TT | 29 | 51.32 | (13.33) ^b^ |  |  | 21 | 49.63 | (12.88) ^b^ |  |  | 8 | 55.75 | (14.34) |  |
| rs247617 | CC | 922 | 43.80 | (12.76) ^a^ | **0.0059** |  | 632 | 41.28 | (11.16) ^a^ | **0.0023** |  | 290 | 49.29 | (14.24) | 0.3962 |
|  | CA | 359 | 44.86 | (13.92) ^a^ |  |  | 255 | 42.55 | (11.97) ^a^ |  |  | 104 | 50.53 | (16.56) |  |
|  | AA | 29 | 51.32 | (13.33) ^b^ |  |  | 21 | 49.63 | (12.88) ^b^ |  |  | 8 | 55.75 | (14.34) |  |
| rs173539 | CC | 791 | 43.89 | (12.85) | 0.3321 |  | 544 | 41.44 | (11.40) | 0.2125 |  | 247 | 49.31 | (14.15) | 0.6489 |
|  | CT | 470 | 44.66 | (13.48) |  |  | 325 | 42.17 | (11.28) |  |  | 145 | 50.25 | (16.14) |  |
|  | TT | 49 | 46.28 | (14.46) |  |  | 39 | 44.55 | (14.19) |  |  | 10 | 53.05 | (14.18) |  |
| rs12720926 | AA | 624 | 44.46 | (12.84) ^a^ | **0.0012** |  | 435 | 41.81 | (11.24) ^ab^ | 0.0675 |  | 189 | 50.57 | (14.18) ^ab^ | **0.0109** |
|  | GA | 562 | 43.17 | (13.14) ^a^ |  |  | 391 | 41.25 | (11.48) ^a^ |  |  | 171 | 47.56 | (15.47) ^a^ |  |
|  | GG | 109 | 48.12 | (14.17) ^b^ |  |  | 73 | 44.64 | (12.46) ^b^ |  |  | 36 | 55.18 | (14.96) ^b^ |  |
| rs7205804 | GG | 608 | 44.48 | (12.79) ^a^ | **0.0052** |  | 425 | 41.86 | (11.08) | 0.1917 |  | 183 | 50.58 | (14.35) ^ab^ | **0.0228** |
|  | GA | 586 | 43.39 | (13.26) ^a^ |  |  | 407 | 41.44 | (11.76) |  |  | 179 | 47.83 | (15.29) ^a^ |  |
|  | AA | 109 | 47.78 | (14.13) ^b^ |  |  | 71 | 44.13 | (12.56) |  |  | 38 | 54.58 | (14.54) ^b^ |  |
| rs711752 | GG | 470 | 44.11 | (12.62) | 0.2006 |  | 328 | 41.86 | (11.42) | 0.2317 |  | 142 | 49.32 | (13.70) | 0.8531 |
|  | GA | 633 | 43.88 | (13.46) |  |  | 443 | 41.36 | (11.57) |  |  | 190 | 49.75 | (15.60) |  |
|  | AA | 207 | 45.74 | (13.27) |  |  | 137 | 43.28 | (11.39) |  |  | 70 | 50.55 | (15.32) |  |
| ***ABCA1*** |  |  |  |  |  |  |  |  |  |  |  |  |  |  |  |
| rs1883025 | CC | 769 | 44.56 | (12.68) ^a^ | **0.0184** |  | 542 | 42.56 | (11.02) ^a^ | **0.0300** |  | 227 | 49.31 | (14.94) ^a^ | **0.0215** |
|  | TC | 459 | 44.47 | (14.35) ^a^ |  |  | 310 | 41.06 | (12.48) ^ab^ |  |  | 149 | 51.56 | (15.39) ^a^ |  |
|  | TT | 82 | 40.29 | (9.24) ^b^ |  |  | 56 | 39.00 | (9.60) ^b^ |  |  | 26 | 43.07 | (7.87) ^b^ |  |
| rs2575876 | GG | 772 | 44.63 | (12.76) ^a^ | **0.0151** |  | 543 | 42.64 | (11.16) ^a^ | **0.0155** |  | 229 | 49.34 | (14.92) ^a^ | **0.0226** |
|  | AG | 457 | 44.35 | (14.27) ^a^ |  |  | 310 | 40.94 | (12.31) ^b^ |  |  | 147 | 51.54 | (15.44) ^a^ |  |
|  | AA | 81 | 40.20 | (8.87) ^b^ |  |  | 55 | 38.84 | (9.06) ^b^ |  |  | 26 | 43.07 | (7.87) ^b^ |  |

Groups denoted with different letters (a, b) indicate statistically significant differences using Scheffe post hoc analysis.

**Table S4.** Association between *ABCA1* and *CETP* genes and stroke outcomes assessed using mRS at 1-, 3-, and 12-months

|  |  | **mRS at 1 months** | | | |  | **mRS at 3 months** | | | |  | **mRS at 12 months** | | | |
| --- | --- | --- | --- | --- | --- | --- | --- | --- | --- | --- | --- | --- | --- | --- | --- |
| ***CETP*** |  | **≤ 2** | **≥ 3** | **OR^a^(95%CI)** | ***P*-value** |  | **≤ 2** | **≥ 3** | **OR^a^(95%CI)** | ***P*-value** |  | **≤ 2** | **≥ 3** | **OR^a^(95%CI)** | ***P*-value** |
| rs3764261 | CC | 633(71.20) | 207(66.13) | 1.0 |  |  | 647(71.41) | 168(64.86) | 1.0 |  |  | 593(70.60) | 154(69.68) | 1.0 |  |
|  | CA | 237(26.66) | 99(31.63) | 1.31(0.94-1.82) | 0.1100 |  | 240(26.49) | 84(32.43) | **1.45(1.01-2.08)** | **0.0438** |  | 229(27.26) | 61(27.60) | 0.94(0.63-1.41) | 0.7794 |
|  | AA | 19(2.14) | 7(2.24) | 1.11(0.41-3.06) | 0.8339 |  | 19(2.10) | 7(2.70) | 1.11(0.38-3.25) | 0.8534 |  | 18(2.14) | 6(2.71) | 1.35(0.46-3.97) | 0.5851 |
|  | Dominant |  |  | 1.29(0.94-1.78) | 0.1170 |  |  |  | 1.42(1.00-2.02) | 0.0508 |  |  |  | 0.98(0.66-1.43) | 0.8970 |
|  | Recessive |  |  | 1.03(0.38-2.81) | 0.9535 |  |  |  | 0.99(0.34-2.89) | 0.9864 |  |  |  | 1.37(0.47-4.00) | 0.5636 |
| rs183130 | CC | 634(71.32) | 208(66.45) | 1.0 |  |  | 648(71.52) | 169(65.25) | 1.0 |  |  | 595(70.83) | 154(69.68) | 1.0 |  |
|  | CT | 236(26.55) | 98(31.31) | 1.28(0.92-1.78) | 0.1436 |  | 239(26.38) | 83(32.05) | 1.41(0.98-2.02) | 0.0639 |  | 227(27.02) | 61(27.60) | 0.95(0.64-1.42) | 0.7942 |
|  | TT | 19(2.14) | 7(2.24) | 1.11(0.40-3.04) | 0.8443 |  | 19(2.10) | 7(2.70) | 1.10(0.37-3.22) | 0.8667 |  | 18(2.14) | 6(2.71) | 1.35(0.46-3.97) | 0.5837 |
|  | Dominant |  |  | 1.27(0.92-1.75) | 0.1512 |  |  |  | 1.38(0.97-1.96) | 0.0727 |  |  |  | 0.98(0.67-1.44) | 0.9121 |
|  | Recessive |  |  | 1.03(0.38-2.81) | 0.9535 |  |  |  | 0.99(0.34-2.89) | 0.9864 |  |  |  | 0.37(0.47-4.00) | 0.5636 |
| rs247617 | CC | 634(71.32) | 207(66.13) | 1.0 |  |  | 648(71.52) | 169(65.25) | 1.0 |  |  | 595(70.83) | 153(69.23) | 1.0 |  |
|  | CA | 236(26.55) | 99(31.63) | 1.29(0.93-1.79) | 0.1337 |  | 239(26.38) | 83(32.05) | 1.41(0.98-2.02) | 0.0639 |  | 227(27.02) | 62(28.05) | 0.96(0.64-1.43) | 0.8449 |
|  | AA | 19(2.14) | 7(2.24) | 1.11(0.40-3.04) | 0.8413 |  | 19(2.10) | 7(2.70) | 1.10(0.37-3.22) | 0.8667 |  | 18(2.14) | 6(2.71) | 0.36(0.46-3.98) | 0.5792 |
|  | Dominant |  |  | 1.27(0.92-1.76) | 0.1410 |  |  |  | 1.38(0.97-1.96) | 0.0727 |  |  |  | 0.99(0.67-1.46) | 0.9619 |
|  | Recessive |  |  | 1.03(0.38-2.81) | 0.9535 |  |  |  | 0.99(0.34-2.89) | 0.9864 |  |  |  | 1.37(0.47-4.00) | 0.5636 |
| rs173539 | CC | 548(61.64) | 178(56.87) | 1.0 |  |  | 558(61.59) | 149(57.53) | 1.0 |  |  | 508(60.48) | 132(59.73) | 1.0 |  |
|  | CT | 308(34.65) | 123(39.30) | 1.30(0.95-1.78) | 0.1030 |  | 316(34.88) | 98(37.84) | 1.25(0.88-1.76) | 0.2152 |  | 303(36.07) | 81(36.65) | 1.06(0.73-1.53) | 0.7746 |
|  | TT | 33(3.71) | 12(3.83) | 0.28(0.59-2.78) | 0.5365 |  | 32(3.53) | 12(4.63) | 1.49(0.65-3.38) | 0.3452 |  | 29(3.45) | 8(3.62) | 1.08(0.43-2.74) | 0.8676 |
|  | Dominant |  |  | 1.30(0.96-1.76) | 0.0948 |  |  |  | 1.27(0.91-1.77) | 0.1659 |  |  |  | 1.06(0.74-1.52) | 0.7577 |
|  | Recessive |  |  | 1.16(0.54-2.50) | 0.7083 |  |  |  | 1.37(0.61-3.08) | 0.4488 |  |  |  | 1.06(0.42-2.66) | 0.8996 |
| rs12720926 | AA | 20(47.84) | 146(47.25) | 1.0 |  |  | 428(47.87) | 119(46.48) | 1.0 |  |  | 396(47.77) | 114(52.05) | 1.0 |  |
|  | GA | 380(43.28) | 140(45.31) | 1.06(0.78-1.45) | 0.7006 |  | 388(43.40) | 116(45.31) | 1.06(0.76-1.49) | 0.7461 |  | 362(43.67) | 84(38.36) | 0.73(0.50-1.06) | 0.0987 |
|  | GG | 78(8.88) | 23(7.44) | 0.84(0.47-1.50) | 0.5558 |  | 78(8.72) | 21(8.20) | 0.89(0.48-1.65) | 0.7098 |  | 71(8.56) | 21(9.59) | 1.09(0.59-2.02) | 0.7747 |
|  | Dominant |  |  | 1.04(0.77-1.40) | 0.8138 |  |  |  | 1.04(0.75-1.44) | 0.8296 |  |  |  | 0.78(0.55-1.11) | 0.1720 |
|  | Recessive |  |  | 0.82(0.47-1.44) | 0.4911 |  |  |  | 0.87(0.48-1.58) | 0.6430 |  |  |  | 1.25(0.69-2.26) | 0.4544 |
| rs7205804 | GG | 410(46.38) | 140(45.02) | 1.0 |  |  | 418(46.39) | 115(44.75) | 1.0 |  |  | 388(46.41) | 108(49.32) | 1.0 |  |
|  | GA | 398(45.02) | 147(47.27) | 1.14(0.83-1.55) | 0.4197 |  | 408(45.28) | 120(46.69) | 1.09(0.78-1.54) | 0.6107 |  | 378(45.22) | 90(41.10) | 0.82(0.56-1.18) | 0.2814 |
|  | AA | 76(8.60) | 24(7.72) | 0.91(0.51-1.63) | 0.7583 |  | 75(8.32) | 22(8.56) | 0.96(0.51-1.78) | 0.8881 |  | 70(8.37) | 21(9.59) | 1.10(0.59-2.05) | 0.7594 |
|  | Dominant |  |  | 1.11(0.82-1.50) | 0.5112 |  |  |  | 1.08(0.78-1.50) | 0.6449 |  |  |  | 0.87(0.61-1.24) | 0.4356 |
|  | Recessive |  |  | 1.17(0.67-2.04) | 0.5922 |  |  |  | 1.09(0.60-1.98) | 0.7780 |  |  |  | 0.82(0.45-1.50) | 0.5207 |
| rs711752 | GG | 310(34.87) | 115(36.74) | 1.0 |  |  | 316(34.88) | 95(36.68) | 1.0 |  |  | 292(34.76) | 92(41.63) | 1.0 |  |
|  | GA | 430(48.37) | 158(50.48) | 0.97(0.70-1.34) | 0.8501 |  | 439(48.45) | 130(50.19) | 0.89(0.62-1.27) | 0.5172 |  | 404(48.10) | 101(45.70) | **0.68(0.46-0.99)** | **0.0446** |
|  | AA | 149(16.76) | 40(12.78) | 0.66(0.41-1.06) | 0.0866 |  | 151(16.67) | 34(13.13) | 0.67(0.40-1.12) | 0.1265 |  | 144(17.14) | 28(12.67) | **0.55(0.32-0.96)** | **0.0342** |
|  | Dominant |  |  | 0.89(0.65-1.21) | 0.4519 |  |  |  | 0.83(0.59-1.17) | 0.2868 |  |  |  | **0.64(0.45-0.92)** | **0.0167** |
|  | Recessive |  |  | 0.67(0.43-1.04) | 0.0747 |  |  |  | 0.72(0.45-1.15) | 0.1667 |  |  |  | 0.69(0.41-1.14) | 0.1467 |
| ***ABCA1*** |  |  |  |  |  |  |  |  |  |  |  |  |  |  |  |
| rs1883025 | CC | 518(58.27) | 194(61.98) | 1.0 |  |  | 528(58.28) | 156(60.23) | 1.0 |  |  | 491(58.45) | 123(55.66) | 1.0 |  |
|  | CT | 325(36.56) | 93(29.71) | **0.62(0.45-0.87)** | **0.0054** |  | 332(36.64) | 75(28.96) | **0.61(0.43-0.89)** | **0.0090** |  | 300(35.71) | 81(36.65) | 1.00(0.69-1.45) | 0.9924 |
|  | TT | 46(5.17) | 26(8.31) | 1.51(0.84-2.72) | 0.1668 |  | 46(5.08) | 28(10.81) | **2.16(1.19-3.91)** | **0.0116** |  | 49(5.83) | 17(7.69) | 1.27(0.63-2.58) | 0.5081 |
|  | Dominant |  |  | **0.72(0.53-0.99)** | **0.0405** |  |  |  | 0.78(0.56-1.09) | 0.1390 |  |  |  | 1.04(0.73-1.47) | 0.8505 |
|  | Recessive |  |  | **1.80(1.01-3.19)** | **0.0453** |  |  |  | **2.58(1.44-4.61)** | **0.0014** |  |  |  | 1.27(0.64-2.54) | 0.4971 |
| rs2575876 | GG | 520(58.49) | 195(62.30) | 1.0 |  |  | 529(58.39) | 158(61.00) | 1.0 |  |  | 492(58.57) | 124(56.11) | 1.0 |  |
|  | GA | 324(36.45) | 91(29.07) | **0.62(0.45-0.87)** | **0.0055** |  | 333(36.75) | 72(27.80) | **0.59(0.41-0.85)** | **0.0048** |  | 299(35.60) | 80(36.20) | 0.99(0.68-1.43) | 0.9425 |
|  | AA | 45(5.06) | 27(8.63) | 1.58(0.88-2.84) | 0.1243 |  | 44(4.86) | 29(11.20) | **2.25(1.24-4.08)** | **0.0078** |  | 49(5.83) | 17(7.69) | 1.19(0.58-2.43) | 0.6331 |
|  | Dominant |  |  | **0.73(0.54-1.00)** | **0.0466** |  |  |  | 0.76(0.54-1.06) | 0.1078 |  |  |  | 1.01(0.71-1.44) | 0.9367 |
|  | Recessive |  |  | **1.88(1.06-3.32)** | **0.0312** |  |  |  | **2.72(1.52-4.88)** | **0.0008** |  |  |  | 1.20(0.60-2.40) | 0.6147 |

a: Adjustment variable: age, gender, BMI, initial stroke severity, hypertension, diabetes, smoking, alcohol drinking, Lipid-lowering drug, and anti-diabetics drug

Dominant model presents that having one or more copies of the major allele increases risk compared to minor allele

Recessive model presents that having two copies of the major allele increases risk compared to one or more copies of minor allele

**Table S5.** Association between combined *ABCA1* rs2575876 variants and HDL-C levels and unfavorable outcomes at 1, 3 and 12 months

|  | **HDL-C** | **ABCA1 rs2575876** | **mRS < 3** | **mRS ≥ 3** | **OR^a^ (95% CI)** | ***P*-value** |
| --- | --- | --- | --- | --- | --- | --- |
| **1 month** | Normal | GG+GA | 264(29.70) | 70(22.36) | 1.0† |  |
|  | Normal | AA | 15(1.69) | 5(1.60) | 1.30(0.39-4.30) | 0.6688 |
|  | Abnormal | GG+GA | 580(65.24) | 216(69.01) | 1.57(1.10-2.25) | 0.0129 |
|  | Abnormal | AA | 30(3.37) | 22(7.03) | 3.10(1.55-6.19) | 0.0014 |
|  |  | RERI |  |  | 1.23 (-5.57-8.02) | |
|  |  | AP |  |  | 0.40 (-0.54-1.33) | |
|  |  | S index |  |  | 2.41 (0.55-10.51) | |
| **3 months** | Normal | GG+GA | 265(29.25) | 57(22.01) | 1.0† |  |
|  | Normal | AA | 13(1.43) | 6(2.32) | 2.42(0.75-7.78) | 0.1339 |
|  | Abnormal | GG+GA | 597(65.89) | 173(66.80) | 1.49(1.01-2.21) | 0.0446 |
|  | Abnormal | AA | 31(3.42) | 23(8.88) | 3.76(1.84-7.68) | 0.0003 |
|  |  | RERI |  |  | 0.84(-6.17-7.86) | |
|  |  | AP |  |  | 0.22(-0.87-1.32) | |
|  |  | S index |  |  | 1.44(0.59-4.53) | |
| **12 months** | Normal | GG+GA | 245(29.17) | 47(21.27) | 1.0† |  |
|  | Normal | AA | 14(1.67) | 4(1.81) | 1.48(0.38-5.77) | 0.4271 |
|  | Abnormal | GG+GA | 546(65.00) | 157(71.04) | 1.80(1.17-2.74) | 0.0273 |
|  | Abnormal | AA | 35(4.17) | 13(5.88) | 1.86(0.80-4.28) | 0.2299 |
|  |  | RERI |  |  | -0.42(-2.72-1.89) | |
|  |  | AP |  |  | -0.23(-2.26-1.81) | |
|  |  | S index |  |  | 0.67(0.03-14.20) | |

Subjects were divided into normal and abnormal groups according to sex and HDL-C level. Normal group (M: 1.04 mM/L≦HDL-C<1.30 mM/L, F: 1.30 mM/L≦HDL-C<1.55 mM/L) and abnormal group ( M: HDL-C<1.04 mM/L or ≧1.30 mM/L, F: HDL-C<1.30 mM/L or ≧1.55 mM/L).

a: Adjustment variable: age, gender, BMI, initial stroke severity, hypertension, diabetes, smoking, alcohol drinking, Lipid-lowering drug, and anti-diabetic drug

†: *P* for trend<0.05

RERI: Relative excess risk due to interaction

AP: Attributable proportion

S index: Synergy index


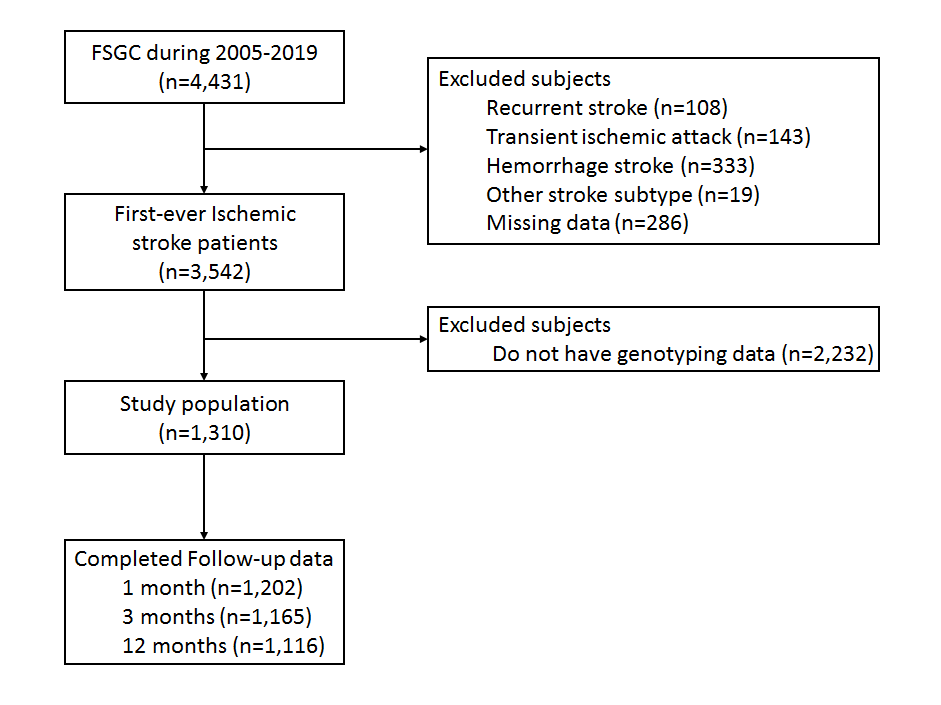


Fig S1. Flowchart of participants recruited for this study


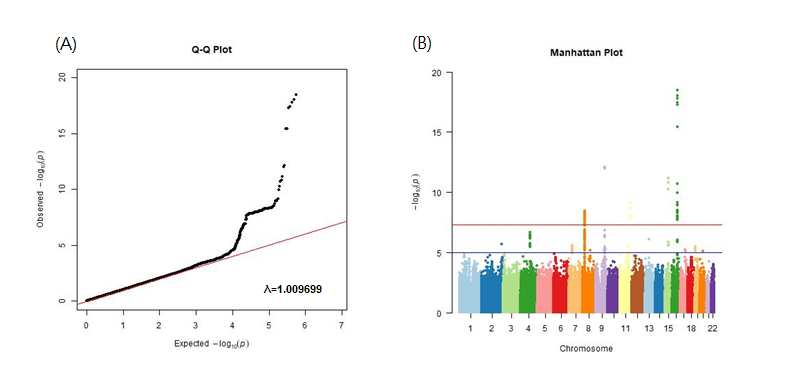


**Fig S2.** (A) Q-Q plot for *P* values from Taiwan Biobank GWAS database. The vertical and horizontal axes indicate observed and expected –log_10_ (*P* value) for tests of association between SNPs and HDL-C level respectively. (B) Manhattan plot (-log_10_ of the *P* value based on genomic location) of the association between the SNPs denoted in the GWAS and the HDL-C levels shows the formation of 15 SNPs over the line representing *P* < 5*10^-8^. GWAS, genome-wide association study; SNPs, single nucleotide polymorphisms; HDL-C, high-density lipoprotein cholesterol


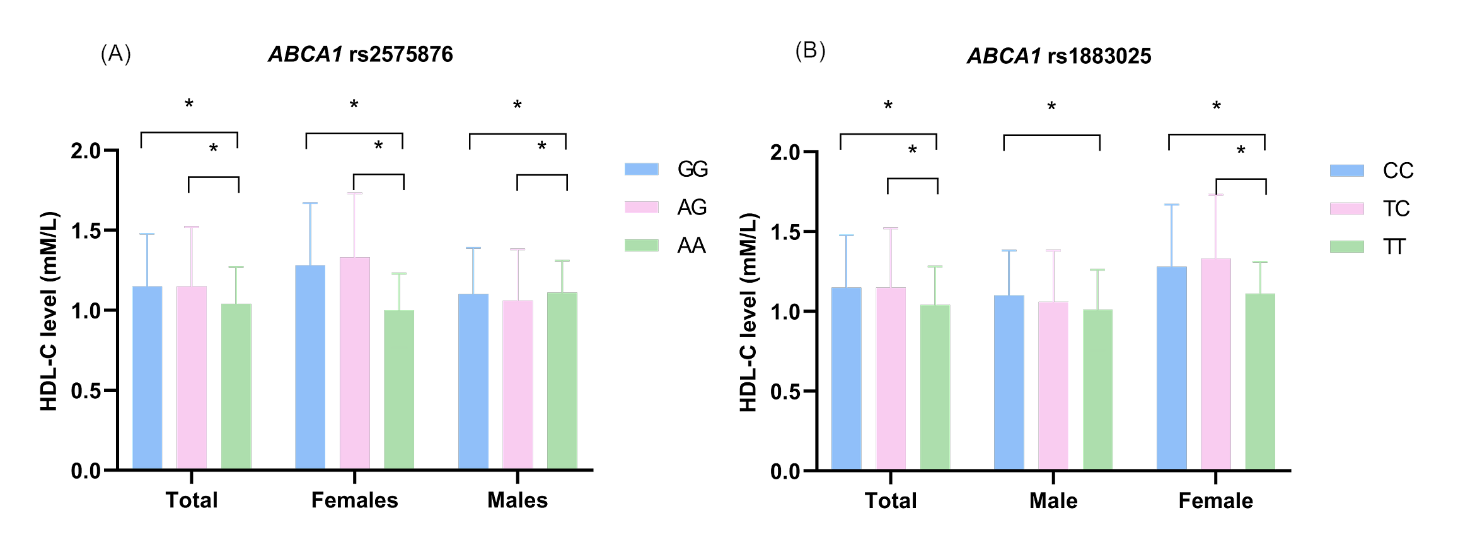


**Fig S3.** Comparison of HDL-C level in each genotype of (A) rs2575876 and (B) rs1883025 SNPs in *ABCA1* gene. HDL-C, high-density lipoprotein cholesterol; SNPs, single nucleotide polymorphisms; ATP Binding Cassette Subfamily A Member 1, ABCA1; *: *P*-value<0.05
